# Mantle cloaking due to ideal magnetic dipole scattering

**DOI:** 10.1038/s41598-020-59291-x

**Published:** 2020-02-12

**Authors:** Barbara Cappello, Anar K. Ospanova, Ladislau Matekovits, Alexey A. Basharin

**Affiliations:** 1 Politecnico di Torino, Department of Electronics and Telecommunications, 10129 Torino, Italy; 2 National University of Science and Technology (MISiS), The Laboratory of Superconducting metamaterials and Department of Theoretical Physics and Quantum Technologies, 119049 Moscow, Russia; 3 Scientific and Technological Center of Unique Instrumentation (RAS), 117342 Moscow, Russia

**Keywords:** Electrical and electronic engineering, Metamaterials

## Abstract

One of the most exciting applications of metaparticles and metasurfaces consists in the *magnetic light* excitation. However, the principal limitation is due to parasitic extra multipoles of electric family excited in magnetic dipole meta-particles characterized by a radiating nature and corresponding radiating losses. In this paper, we propose the “*ideal magnetic dipole*” with suppressed additional multipoles except of magnetic dipole moment in the scattered field from a cylindrical object by using mantle cloaking based on metasurface and on anapole concept. The considered metasurface consists of a periodic width modulated microstrip line, with a sinusoidally shaped profile unit cell printed on a dielectric substrate.

## Introduction

Despite of the progress in electromagnetics, the light-matter interaction is still associated especially with electric component of light, while magnetic component is suppressed down to negligible level, particularly at more anticipated optical frequencies. On the other hand, *magnetic light-matter interaction* is promising playground for unusual effects like negative refraction^1–4^, magnetoinductive waves^5^, fluorescent microscopy^6,7^, nanoscale imaging and others^8,9^. Therefore, magnetic response of subwavelength particles contributes to optical magnetism leading to effects such as magnetic light^10^, magnetic nanoantennas^11–15^ and magnetic Purcell effect^16–19^. For this sake, recent works propose various techniques for artificial magnetism that enable strong magnetic field localization even for nonferromagnetic particles that is usually several times weaker than the electric field component counterpart^20–24^. The first realization of artificial magnetic dipole (MD) has been demonstrated in metal split-ring resonator (SRR), thereafter becoming a basic element of metamaterials^3,25,26^. Overall, strongly concentrated magnetic field can be excited by a circular current oscillating within the SRR and mimicking a MD. The idea of SRR is still of high demand; however, its application is restricted by the impossibility of implementation in visible frequency range due to intrinsic metallic particles with high Joule losses^26^. A second limitation is due to the excitation of parasitic extra multipoles of electric family characterized by a radiating nature and corresponding radiating losses. Thus, researchers are approaching to achieve an *ideal magnetic dipole* without additional multipoles (except of magnetic dipole moment) in the system.

The idea of the *ideal MD scatterer* can be elegantly described by multipole decomposition theory of the scattering from resonant particles. This approach allows studying the radiation properties by description of the scattering cross-section as a sum of electric *a*_*E*_*(l, m)* and magnetic *a*_*M*_*(l, m)* scattering coefficients. Indeed, the resulting scattering efficiency *Q*_*sca*_ is given by the superposition of electric and magnetic scattered multipoles:

1 $${Q}_{sca}=\frac{\pi }{{k}^{2}}\mathop{\sum }\limits_{l=1}^{\infty }\mathop{\sum }\limits_{m=-l}^{l}(2l+1)({|{a}_{E}(l,m)|}^{2}+{|{a}_{M}(l,m)|}^{2})$$

where *l* is the number of spherical harmonics defining multipoles order. In this way, we can calculate the scattering intensity of electric multipoles described by the first term and of magnetic multipoles, expressed as second term in Eq. (1), directly through the induced currents inside the particles^27^. Inspired by the *ideal MD scatterer* approach, we should find the current distribution with electric multipoles *a*_*E*_*(l, m)* scattering intensity tending to zero. In this case, *Q*_*sca*_ is almost described by the second term in Eq. (1), i.e., *a*_*M*_*(l, m)*, given by the magnetic dipole mode. One of the elegant solutions is anapole mode excitation, defined as electric type scattering elimination by toroidal dipole moment at the same frequency^28,29^. Consequently, the spectral overlapping of magnetic and anapole modes provides the pure MD scattering in far-field zone. This concept has recently been introduced by Feng *et al*.^30^ for complex high refractive index core-shell nanoparticles.

In this work, we propose the metal-dielectric hybrid design for *ideal MD scatterer* with eliminating electric type scattering due to anapole mode excitation. Therefore, the operating frequency corresponds to the minimum radar cross section (RCS) and, additionally, it demonstrates collateral mantle cloaking effect.

The *anapole mode* interpretation requires the introduction of the *toroidal multipoles* – a separate family of multipoles of complex electromagnetic configuration^31,32^. Let us imagine the poloidal currents ***j*** flowing along the torus meridians and generating a magnetic field ***m*** in metaparticles of toroidal shape. Respectively, the toroidal dipole (TD) moment appears as an oscillation along the torus main axis and manifests a strong field localization even within the point-like source, possessing far-field radiation pattern similar to the electric dipole (ED) mode. Namely, the interference between ED and TD modes under the condition **P** = *ik***T**, where **P** stands for ED moment and **T** is the TD moment, mutually cancels their far-field radiations^29^. This condition, also called anapole mode, leads to nonradiating states intended for development of nonradiating sources, noninvasive sensors, cloaking devices, etc.^33–47^.

The multipole decomposition of far-field intensities that includes toroidal multipoles contribution is presented in the work by Vaman and Radescu^48^ and are expressed as:

1a $$I=\frac{2}{3}\frac{{\omega }^{4}}{{c}^{3}}{|{\bf{P}}|}^{2}+\frac{2}{3}\frac{{\omega }^{4}}{{c}^{3}}{|{\bf{M}}|}^{2}+\frac{4}{3}\frac{{\omega }^{5}}{{c}^{4}}Im({\bf{P}}\dagger {\bf{T}})+\frac{2}{3}\frac{{\omega }^{6}}{{c}^{5}}{|{\bf{T}}|}^{2}$$

Where *c* denotes the speed of light, *ω* is the angular frequency and the symbol † indicates the Hermitian operator.

Indeed, in the case **P** = *ik***T**, one can conclude that the previous equation will transform to:

1b $$\begin{array}{ccc}I({\boldsymbol{P}}=ik{\boldsymbol{T}}) & = & \frac{2}{3}\frac{{\omega }^{6}}{{c}^{5}}{|{\bf{T}}|}^{2}+\frac{2}{3}\frac{{\omega }^{4}}{{c}^{3}}{|{\bf{M}}|}^{2}+\frac{4}{3}\frac{{\omega }^{5}}{{c}^{5}}Im(-{\rm{ik}}{|{\bf{T}}|}^{2})+\frac{2}{3}\frac{{\omega }^{6}}{{c}^{5}}{|{\bf{T}}|}^{2}\\  & = & \frac{2}{3}\frac{{\omega }^{6}}{{c}^{5}}{|{\bf{T}}|}^{2}-\frac{2}{3}\frac{{\omega }^{6}}{{c}^{5}}{|{\bf{T}}|}^{2}+\frac{2}{3}\frac{{\omega }^{4}}{{c}^{3}}{|{\bf{M}}|}^{2}=\frac{2}{3}\frac{{\omega }^{4}}{{c}^{3}}{|{\bf{M}}|}^{2}\end{array}$$

Thus, the system characterized by anapole (**P** = *ik***T**) state accompanied by the presence of magnetic dipole **M** will scatter as an ideal MD, since only that component is present on the right hand side in the equation above.

## Results

### The metasurface design

For the realization of the *ideal MD scatterer* concept, here we propose the design of a structure consisting of an infinitely elongated Perfectly Electric Conductor (PEC) cylinder core and a hybrid PEC metasurface coating with a dielectric layer embedded between them. Providing that the PEC core is a strong electromagnetic scatterer, the dielectric coating with imprinted metasurface of sinusoidal-like pattern is assumed to considerably reduce its scattering properties. From electromagnetic point of view, a metasurface can be considered as a mantle cloaking device^49–51^, since cloaking can be realized by the use of a thin layer usually composed of periodic arrangement of unit cells in 2D. Thus, we treat this structure based on its scattering properties and estimate the field distribution properties^52–54^.

The characteristics of the metasurface can be equivalently defined in terms of surface impedance. This latter modifies the boundary condition of the object connecting the incident tangential electric field with the induced surface currents^51,55^.

Considering a PEC cylinder, the total field, in cylindrical coordinates, can be expressed as an infinite sum of harmonics in the form:

2 $${E}_{z}(\rho ,\phi )={E}_{0}\mathop{\sum }\limits_{m=-\infty }^{+\infty }{i}^{-m}\,[{J}_{m}({k}_{b}\rho )+{c}_{m}{H}_{m}^{(2)}({k}_{b}\rho )]{e}^{im\phi }\,\rho  > b$$

where *J*_*m*_ and ${H}_{m}^{(2)}$are Bessel and Hankel functions that describe the incident and the scattered field, respectively, *c*_*m*_ are the scattering coefficients, *k*_*b*_ is the wavenumber in the background medium that here is considered as vacuum.

Scattering cancellation can be therefore achieved by the annulment of coefficients *c*_*m*_. This leads to the formulation of *Z*_*s*_(*m*) = 1/*Y*(*m*) as expressed in^56^, where $${Y}_{s}(m)=-\,i\cdot {J{\prime} }_{m}({k}_{b}b)/{J}_{m}({k}_{b}b)\,+i\sqrt{{\varepsilon }_{r}}\cdot [{H}_{m}^{(2){\prime} }(kb)+\gamma (ka){H}_{m}^{(1){\prime} }$$$$(kb)]/[{H}_{m}^{(2)}(kb)+\gamma (ka){H}_{m}^{(1)}(kb)]$$, being *a* and *b* the (external) radius of the metallic cylinder without and with coating, respectively. In the previous expression “prime” denotes the derivative (with respect to the radial coordinate *ρ*) and $$\gamma (ka)=-\,{H}_{m}^{(2)}(ka)/{H}_{m}^{(1)}(ka)$$.

In this way, it can be proven that a single value of the surface impedance is directly associated to one harmonic index *m* and therefore to the annulment of a single scattering coefficient (usually the dominant one, if it does exist).

Practically, the surface impedance layer is realized using a 2D metasurface formed by the periodical replication of a given unit cell. The surface impedance value, and therefore the RCS suppression frequency, can be controlled by acting on the geometrical parameters of the metasurface unit cell. Recently, different types of unit cells, such as patches, strips, crosses, have been used^49–51,56–64^.

In this paper we consider a non-electrically small cylinder. In this case, a larger number of harmonics contribute to the scattered field^49^; therefore, a homogeneous surface impedance is not sufficient to cloak the cylinder. For this reason, in the following we propose a modulated cell profile, in order to provide a non-homogeneous value of surface impedance and therefore to enlarge the cloaking bandwidth. The expected bandwidth enlargement can also be explained by the fact that, similar to biconical structure, the modulated profile incorporates different resonant lengths of closely located frequencies that give rise to a wide-band resonance.

**Figure 1:**
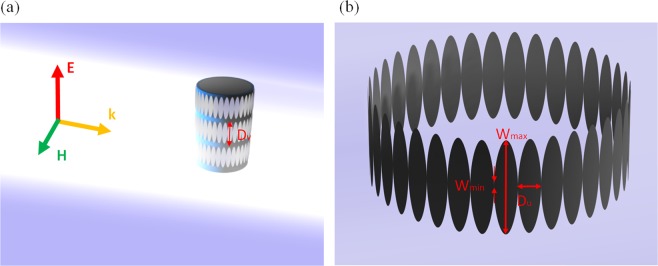
Illustration of the proposed metallic-dielectric coating dressed on metallic cylinder (**a**). Resulting pattern of metasurface rings and geometrical parameters (**b**).

In particular, the structure consists of a PEC cylinder of radius *r*_PEC_ = a = 20 mm wrapped into a hybrid coating composed of a dielectric substrate of relative permittivity *ε*_*r*_ = 3, for an easier practical realization, and an infinitely thin metasurface layer (let us suppose PEC layer) (Fig. 1a). The thickness of the dielectric substrate is *t* = 2.92 mm and its height is *D*_*v*_ = 32 mm.

For convenient field distribution estimation, we introduce the cell profile described by the analytical formulation in^54^:

3 $$W(u)=\frac{{W}_{{\min }}}{2}+\frac{({W}_{{\max }}-\,{W}_{{\min }})}{2}{({\sin }\frac{\pi u}{{D}_{u}})}^{\alpha }$$

where the parameter *α* is related to the cell modulation that ensures a variation of the dielectric constant and the offered surface impedance inside the unit cell leading, therefore, to an enlargement of the operation bandwidth^52–54^.

The unit cell period is determined by the length *D*_*u*_ = 4 mm, while the minimum and maximum height of metallic pattern are *W*_min_ = 0.2 mm and *W*_max_ = 27.3 mm, respectively. The one-round wrapping contains *N* = 36 unit cells (Fig. 1b). During the numerical simulations, performed with commercial solver Microwave Studio by CST, periodic boundary conditions along the ±*z* directions have been set, imitating an infinitely long cylinder. In such a way, no edge diffraction effects are present in the results that could act as noise in the analysis described below.

Variations of the unit cell parameters enable to control the operation frequency of the scatterer. In particular, the working frequency is inversely proportional to *W*_max_^54^.

### Scattering properties and field distribution

To study the ideal magnetic dipole scatterer, we consider the scattering properties by computing the object RCS and the field maps of the electric and magnetic field components intensities. Moreover, we estimate the evolution of the chosen parameters and we study the electromagnetic response of scatterers with similar pattern of a different ellipse height *W*_*max*_, namely, *W*_1_ = 27.3 mm, *W*_2_ = 23.4 mm, *W*_3_ = 19.5 mm, *W*_4_ = 17.5 mm and *W*_5_ = 15.6 mm, while maintaining *D*_*v*_ and *W*_*min*_ constant.

**Figure 2:**
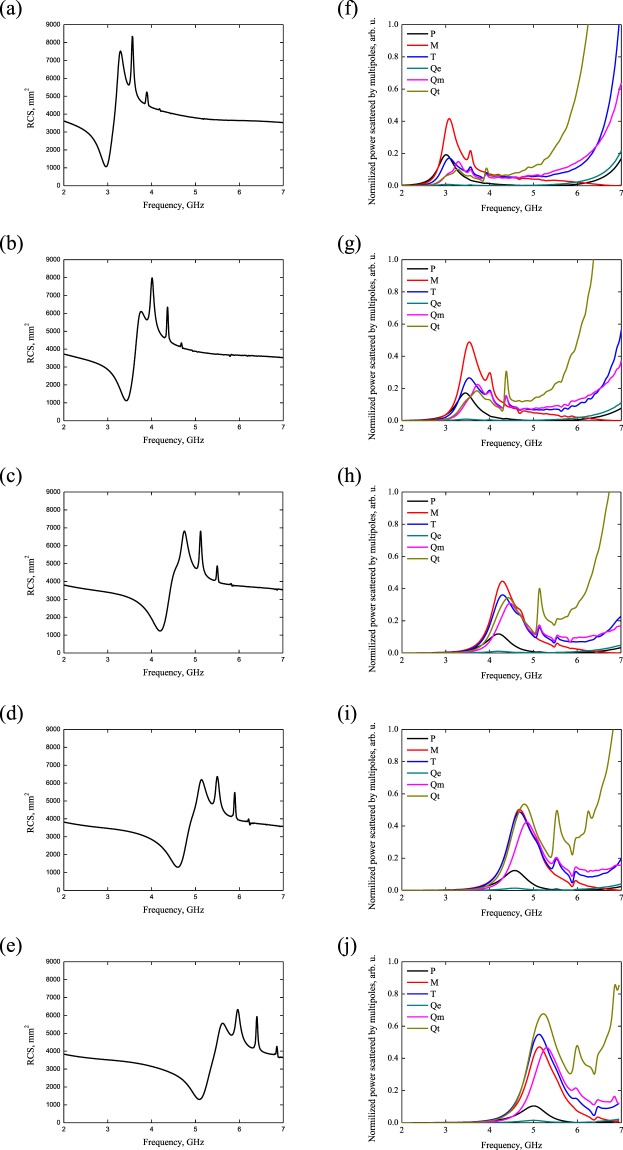
RCS patterns for different metallization height, (**a**) *W*_1_ = 27.3 mm, (**b**) *W*_2_ = 23.4 mm, (**c**) *W*_3_ = 19.5 mm, (**d**) *W*_4_ = 17.5 mm and (**e**) *W*_5_ = 15.6 mm. Normalized power scattered by multipoles for different ellipse height of coating, (**f**) *W*_1_ = 27.3 mm, (**g**) *W*_2_ = 23.4 mm, (**h**) *W*_3_ = 19.5 mm, (**i**) *W*_4_ = 17.5 mm and (**j**) *W*_5_ = 15.6 mm.

The structure is illuminated by a normally incident plane wave of TM polarization and it shows minimum RCS (~1000 mm^2^) for *W*_1_ = 27.3 mm at *f* = 2.96 GHz (Fig. 2a). As for the other patterns of the metasurface, the RCS is higher up to 1200 mm^2^ and shifted toward higher frequencies (Fig. 2b–e). Moreover, strong oscillations at higher than the resonance frequencies correspond to a resonance behavior of scattering of the system.

**Figure 3:**
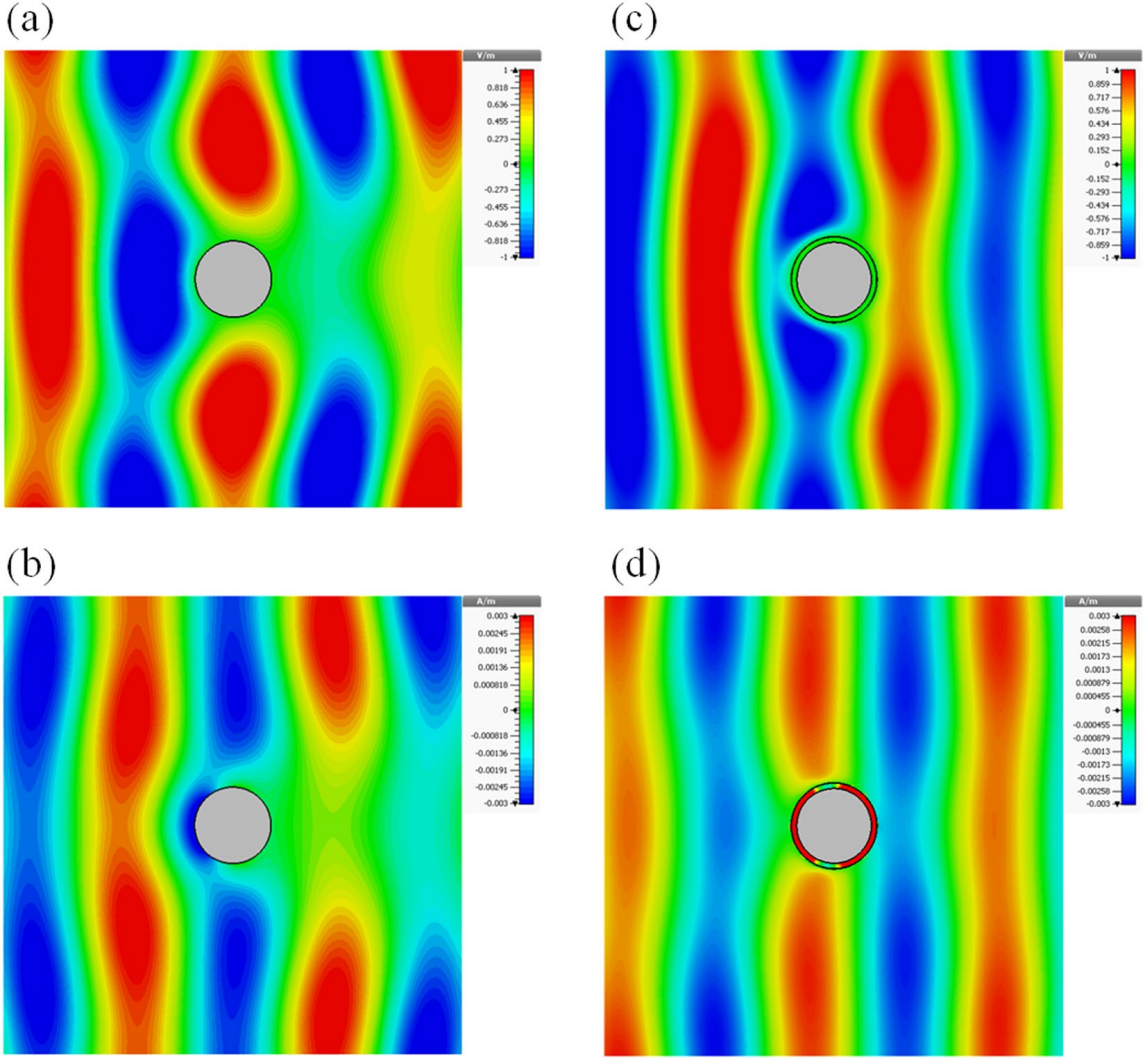
Electric (**a**,**c**) and magnetic (**b**,**d**) fields of the bare (left) and cloaked (right) cylinder with *W*_*max*_ = 27.3 mm at *f* = 2.96 GHz.

Furthermore, we study the electric and magnetic field distribution at the minimum scattering frequency *f* = 2.96 GHz for cylinder coated with PEC metasurface (Fig. 3c,d) and for bare PEC cylinder at the same frequency (Fig. 3a,b). In the case of the bare cylinder, electric and magnetic field amplitude distributions evidently resemble the scattering from simple electric scatterer. On the other hand, it can be observed that the field properties of the metasurface coated PEC cylinder exhibit a cloaking effect. Indeed, the electric and magnetic field perturbations are strongly reduced, and field lines replicate the plane wave propagation.

**Figure 4:**
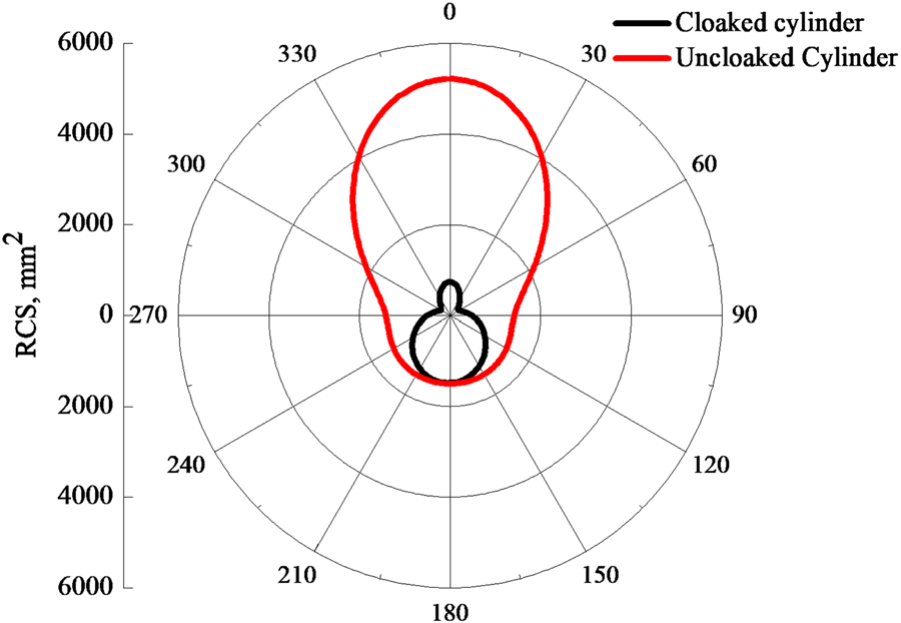
Far-field scattering of the bare and cloaked cylinder with *W*_*max*_ = 27.3 mm at *f* = 2.96 GHz.

Moreover, we compare far-field scattering of the bare and the cloaked cylinder. The results are reported in Fig. 4, showing a reduction of the RCS for all directions around the cylinder. Further details on the cloaking effect of the metasurface such as the dependence on the materials properties or the scalability of the structure are reported in the Supplementary material.

### The multipolar description of near-field interaction

The origin of the suppressed RCS and accompanied mantle cloaking effect can be accurately explained in terms of multipole decomposition approach. We have studied the near-field properties of the interaction between incident TM-polarized wave and ideal MD scatterer. In this instance, multipolar decomposition provides an accurate description of the near-field properties due to charge-current redistribution inside the object^48^ and interpreted far-field scattering fields. The lowest order multipoles, such as dipoles and quadrupoles, are considered as the strongest ones and are related to electric, magnetic and toroidal families. This selection of multipoles correctly characterizes the pseudotorus topological electromagnetic excitations^29,31,47^.

For explanation of the origin of the encountered cloaking effect, we have carried out multipolar decomposition near the minimum scattering value at *f* = 2.96 GHz (Fig. 2f). The scattering intensity of the strongest multipoles is given in terms of electric **P**, magnetic **M** and toroidal **T** dipoles, electric **Q**e, magnetic **Q**m and toroidal **Q**t quadrupoles. The key role in the scattering characterization is played by the magnetic dipole mode **M** which dominates all other standard multipoles (Fig. 2f). In the proximity of the resonance frequency, the power scattered by the magnetic dipole moment is more than 2 times higher than that of the electric and toroidal dipole moments and corresponding quadrupoles. However, this pronounced scattering resonance is due to the suppression of the far-field scattering defined by electric-type of multipoles – we remind here that electric and toroidal radiations exhibit identical radiation patterns. More significantly, minimal scattering at *f* = 2.96 GHz is accompanied by the overlapping of electric **P** and toroidal **T** dipole moments resulting in anapole mode excitation, more appreciated for total elimination of the electric scattering due to the appearance of the **P** = *ik***T** condition. That said, the scattering origin is interpreted as a magnetic response of higher magnetic dipole intensity associated with the fully eliminated electric component of scattering.

We also note that the initial response of PEC cylinder is due to electric dipole mode with the suppression of other multipoles contribution, including magnetic response. On the other hand, the metasurface plays the role of cloak that could be described as a compensating element, having influence only on electric types of multipoles. Thus, the cloaking technique accompanied by a RCS minimum is also determined by a magnetic scattering response, hence the cancelation of electric scattering coefficient in Eq. (1). In this view, we consider the different parameters of the metasurface. Even if the magnetic dipole moment has the maximum intensities for *W*_*max*_ = 23.4 mm and *W*_*max*_ = 19.5 mm, anapole mode cannot be established due to unfulfillment of the **P** = *ik***T** relation, i.e., the toroidal mode is shifted in frequency from the resonance for these cases. Thus, the RCS achieves 1193 mm^2^ and 1233 mm^2^ for *W*_*max*_ = 23.4 mm and *W*_*max*_ = 19.5 mm, respectively. However, the toroidal quadrupole **Q**t plays the key role at resonance appearing in cases of *W*_*max*_ = 17.5 mm and *W*_*max*_ = 15.6 mm.

It should be stressed that the RCS damping by anapole mode is crucial to realize a magnetic dipole scattering by the cylindrical particle for a TM-polarized wave excitation. Our results demonstrate that the proposed nonmagnetic metaparticle can be used for magnetization without complex systems providing currents circulation as in SRR and hybrid nanoparticles.

Therefore, we have demonstrated a novel approach for realization of a mantle cloaking of simplified design for the generation of magnetic light due to ideal magnetic dipole scattering, previously proposed only for hybrid metal-dielectric nanoparticles.

## Discussion

Summarizing, we have presented a cloaking technique appreciated for the number of applications from widely known camouflage and stealth techniques for military purposes to modern medical and biological probes. The interest in cloaking techniques arises due to strong reduction of fields scattered from the cloaked object in all directions around the scatterer position. This leads to undisturbed field interaction between an incident light so that the cloaked object becomes invisible for external observers.

Modern literature reports on various cloaking techniques available due to their simple practical implementation. Apart from fundamentally important transformation optics (TO) technique, the plasmonic cloaking (PC) and mantle cloaking (MC)^49–51,60–62,65,66^ are more common from a practical point of view. In literature, different approaches to control the scattering of light are found^67–77^. In particular, MC differs from others since it does not require bulk anisotropic materials and is realized by using a thin layer metasurface usually consisting of a periodic arrangement of unit cells. Moreover, we considered the cloaked device in terms of magnetic response, discussed in previous literature to enhance the Purcell effect^78^, and anapole mode^79,80^. In our analysis, the cloaking effect is explained by the suppression of scattering multipoles of the same polarization as of the incident wave (electric and toroidal dipole moment). Additionally, magnetic dipole-like response has been demonstrated that is due to the excess part of the anapole effect of electric type.

## Conclusion

In this paper, we considered a new type of ideal magnetic metal-dielectric hybrid scatterer based on well-pronounced magnetic dipole moment with simultaneously suppressed electric response leading to minimization of total scattering. Therefore, this strong scattering suppression has a similar consequence as mantle cloaking effect; at the specific resonance frequency, one could describe the cloaking effect due to multipoles interplay. Moreover, we proposed a properly design metasurface for practical realization of ideal magnetic dipole effect. Its performances have been numerically proved.

## Methods

### Simulations

The electromagnetic properties of metal-dielectric hybrid design is computed by commercial Maxwell’s equation solver, namely, CST Microwave Studio, using the standard transient modeling approach. The simulations provide values of RCS, giving data on scattering properties of the structure. Furthermore, the field maps of electric and magnetic field distributions in near-field of the structure are obtained. The current densities induced in the structure are used to calculate the powers radiated by conventional multipoles, including those of toroidal dipoles.

## Supplementary information

Supplementary Material.
