# Supplementary material for: Mantle cloaking due to ideal magnetic dipole scattering
Source: Sci Rep. 2020 Feb 12;10:2413. doi: 10.1038/s41598-020-59291-x (PMC7016144; doi:10.1038/s41598-020-59291-x)
Supplement: Supplementary file 1 — Supplementary Material. [file 41598_2020_59291_MOESM1_ESM.docx]

**Mantle cloaking due to ideal magnetic dipole scattering**

Barbara Cappello^1^, Anar K. Ospanova^2, 3^, Ladislau Matekovits^1^, Alexey A. Basharin^2, 3,*^

^1^Politecnico di Torino, Department of Electronic and Telecommunications, 10129, Torino, Italy

^2^National University of Science and Technology (MISiS), The Laboratory of Superconducting metamaterials and Department of Theoretical Physics and Quantum Technologies, 119049, Moscow, Russia

^3^Scientific and Technological Center of Unique Instrumentation (RAS), 117342, Moscow, Russia

**Supplementary material**

This file incorporates some of the issues that are pertinent to the mainstream discussed in the main paper. It includes different topics that are presented as separate sections.

In order to estimate different features of the cloaking structure, we provide information about different parameters of the system.

1. Fano Fitting

The RCS spectra can be defined by Fano curve. In order to estimate the Q-factor of cloaking, we have performed the Fano fitting of the RCS response, exploiting the following expression:

$$RCS\left( f \right)= A_{0}+\frac{C\left( F\gamma+\frac{f}{f_{n}}-1 \right)}{\left( \frac{f}{f_{n}}-1 \right)^{2}+\gamma^{2}}$$

where RCS offset $A_{0}=$1000, normalized constant $C=$2900, normalized frequency $f_{n}=$3.1 GHz, Fano parameter $F=$0.9583, normalized linewidth of resonance $\gamma=$55.18e-3, have been considered.

The result in quality Q- factor is $Q=1/\gamma$=18.123.

Figure S1. Fano fitting

1. Scalability and use of non-ideal materials.

Indeed, the real metals instead of PEC can reduce the performance of cloaking. However, we simulate the structure substituting the previously considered PEC with gold. The results of the RCS do not show significant differences with respect to the ideal PEC case. Moreover, the dimensions of the structure have been scaled from mm to μm and it has been simulated in the THz and IR range. Also in this case, the RCS does not show appreciable differences and therefore the scalability of the structure has been proved in THz regime up to 3 THz. However, if we scale the proposed structure up to 10^-6^ times and consider IR optics, the resonance deep is broadening. The results are reported in Figs. S2(a,b,c).

Figure S2b. RCS result for the structure scaled from mm to um. Also in this case gold has been used as conductive material.

Figure S2a. RCS result when gold is used as conductive material.

Figure S2c. RCS result for the structure scaled from mm to nm. Also in this case gold has been used as conductive material.

1. Variation of the dielectric constant.

We can expect that increasing the dielectric constant, the bandwidth of the cloaking structure reduces, since resonators filled with dielectric with higher relative permittivity are intrinsically of narrow band.

In Figs. S3a-S3d, the results of the RCS for different dielectric constants are reported. It can be seen that the frequency of operation is lower and moreover the bandwidth is reduced with respect to the case with a dielectric constant *ε_r_*=3 considered in the main paper. Although, at resonance, the RCS reaches 1000 mm2 as before.

The structure has been simulated considering different values for the relative permittivity of the dielectric layer, namely $\varepsilon_{r}$ = 5,7,9,11.

Figure S3a. RCS results when the relative permittivity of the dielectric layer is set $\varepsilon_{r}=$ 5.

Figure S3b. RCS result when the relative permittivity of the dielectric layer is set $\varepsilon_{r}=$ 7.

Figure S3c. RCS result when the relative permittivity of the dielectric layer is set $\varepsilon_{r}=$ 9.

Figure S3d. RCS results when the relative permittivity of the dielectric layer is set $\varepsilon_{r}=$ 11.

1. Oblique incidence.

For the sake of completeness, we calculate the RCS for different angles of incidence of the exciting wave. The results are reported in Figs. S4(a,b,c). We note, that the cloaking effect and the resonance frequency are stable till an incidence of 30°.

It is important to notice that for this analysis a finite length structure has been considered proving also that the results obtained for infinitely long cylinder are still valid in case of a finite structure. In particular, we simulated the structure considering five unit cells in the longitudinal direction $\hat{z}$.

Moreover, we have observed the following interesting effect: For large angles close to 90° (Fig. S4c) the structure strongly scattered and RCS tends to 35000 mm^2^, instead of cloaking ~1000 mm^2^ on 3 GHz for the infinite cylinder and for normal incidence (Fig. 2a).

Figure S4a. RCS results considering an oblique excitation, $\theta=30^{\circ}$.

Figure S4b. RCS results considering an oblique excitation, $\theta=60^{\circ}$.

Figure S4c. RCS result considering an oblique excitation, $\theta=\sim90^{\circ}$.
